# Faculty Perspectives on Roles and Tensions in Supporting Student Learning and Self-Regulation Through Feedback and Coaching in Programmatic Assessment

**DOI:** 10.5334/pme.1923

**Published:** 2025-10-22

**Authors:** Xian Liu, Diana Dolmans, Maryam Asoodar, Zhien Li, Daniëlle Verstegen

**Affiliations:** 1 Department of Educational Development & Research, School of Health Professions Education, Faculty of Health, Medicine and Life Sciences, Maastricht University, Maastricht, The Netherlands

## Abstract

**Introduction:**

In health professions education, assessment is increasingly viewed not merely as a tool for grading, but also as a means of shaping a productive learning environment. This shift promotes student-centered learning and enhances students’ self-regulated learning (SRL). Consequently, faculty members are expected to take on the role of facilitators, providing rich feedback and coaching to support student learning. However, this is not easy in practice, and these underlying mechanisms are not yet fully understood. The research question of this exploratory study is: How do faculty perceive their roles and the tensions in supporting student learning and self-regulation through feedback and coaching in programmatic assessment?

**Methods:**

This study adopted a qualitative constructivist paradigm. Seventeen faculty members participated in five semi-structured focus group interviews. Thematic analysis was employed, integrating both inductive and deductive approaches.

**Results:**

The analysis produced four key themes: (1) Providing feedback at the task level is easier than giving suggestions for further development of competencies; (2) Tailoring narrative feedback to personalized learning paths is challenging; (3) The primary role of coaching is to foster students’ reflection and autonomy, while adapting to students’ needs when necessary; (4) Coaching requires a balance between stimulating autonomy and fulfilling graduation requirements.

**Discussion:**

This study shows that faculty believe that programmatic assessment enhances learning, but they experience challenges and tensions in stimulating students’ SRL in practice. It is not easy to tailor feedback to meet students’ individual needs and to align coaching for SRL with graduation requirements.

## Introduction

Recently, assessment in health professions education has undergone a fundamental transformation. Assessment is increasingly viewed not merely as a grading tool but as a mechanism for shaping effective learning environments [1]. Programmatic assessment (PA) has emerged as a comprehensive framework that embodies this transformed approach to assessment [2]. PA highlights the potential of assessment to foster student-centered learning and enhance students’ SRL capabilities [3], emphasizing learners’ active role in engaging with and making sense of assessment practices. SRL describes learning as a cyclical process through which students proactively regulate their cognition, motivation, and behavior to achieve academic and professional goals within specific contexts [4,5]. Three interrelated phases (forethought, performance, and self-reflection) describe how learners adapt strategies and monitor progress. SRL is important in health professions education, where the rapid evolution of knowledge necessitates lifelong learning and continuous competency development [6].

This reconceptualization of assessment and learning has significant implications for faculty members, who are now expected to shift from traditional roles as evaluators to facilitators of learning. This shift requires faculty members’ sustained engagement in feedback and coaching practices to guide students’ metacognitive growth and foster ownership of learning [7]. They need to provide rich, formative assessment opportunities to support students’ agency and self-regulatory development [8]. Therefore, PA utilizes continuous feedback and coaching to promote development across competencies, employing multiple low-stakes assessments to inform high-stakes decisions [9]. Rich feedback facilitates ongoing, comprehensive assessments of student performance while promoting learner ownership and SRL [10], while coaching further aids students by helping them make sense of feedback and develop strategies for improvement [11].

Thus, feedback and coaching are key mechanisms to support student learning, as they help students interpret and use assessment information [11]. However, implementation presents considerable challenges. Faculty face time and cognitive demands in delivering meaningful, individualized feedback [12] and often need to interpret complex longitudinal data while managing potentially conflicting messages from multiple assessors [13]. Moreover, they must balance fostering student autonomy with offering appropriate levels of guidance [14].

While the literature underscores the importance of faculty in facilitating student learning and stimulating SRL, there is limited understanding of how faculty themselves enact these evolving roles and what tensions they experience providing feedback and coaching [15]. Gaining deeper insights into these experiences is critical for supporting faculty and optimizing their role in PA. Therefore, this study addresses the following research question: How do faculty perceive their roles and the tensions in supporting student learning and self-regulation through feedback and coaching in programmatic assessment?

## Methods

### Study Design

This qualitative study adopted a constructivist paradigm to explore participants’ experiences with providing feedback and coaching in the PA context, recognizing the socially constructed nature of participants’ experiences and perspectives in an assessment environment. Thematic analysis was used, integrating inductive and deductive approaches to uncover both explicit and underlying patterns in the data [16].

### Setting

We recruited participants from the faculty members of the Master of Health Professions Education (MHPE) program, School of Health Professions Education, Maastricht University. The MHPE program is a primarily online, part-time postgraduate program for healthcare professionals aiming to advance in health professions education. The curriculum employs PA and emphasizes authentic learning tasks, individualized and flexible learning pathways (with 60% electives), narrative feedback, e-portfolio use, and personalized coaching. MHPE students have diverse backgrounds and often balance their studies with clinical or teaching roles in local hospitals. Most faculty members fulfill multiple roles, for example, as feedback providers, coaches, and assessment committee members.

### Participants

Seventeen MHPE faculty members participated in this study. Participants were selected via purposive sampling, ensuring they had at least one year of teaching experience in the MHPE program and familiarity with the entire curriculum, including both online and campus-based components. All 20 eligible faculty members were invited via institutional email, and 17 agreed to participate, providing broad representation of the core teaching team and a mix of seasoned and newer professionals: the majority of faculty (41.2%) had over 15 years of teaching experience, and nearly a third (29.4%) had fewer than five years. Most participants (82.4%) served as both feedback providers and coaches, with only a small proportion fulfilling just one of these roles (11.8% and 5.9%, respectively).

### Data Collection

Five online, semi-structured focus group sessions were conducted. Each session included three to four participants and lasted approximately one hour. The focus group format fostered rich, interactive dialogue, allowing participants to reflect on and compare their experiences collectively.

The focus group interview guide (see Appendix 1) was informed by Zimmerman’s (2002) SRL framework, focusing on how faculty perceive their feedback and coaching supports students’ planning, monitoring, and reflection. The high participation rate (17 of 20 eligible faculty) and the absence of new insights in the analysis of the final transcripts indicate adequate depth and relevance of the data to address the research aim [17].

### Data Analysis

Focus group interviews were recorded, transcribed, and anonymized by the primary researcher (XL) before analysis. Data analysis was guided by Zimmerman’s SRL framework using the three cyclical phases (forethought, performance, and self-reflection) as sensitizing concepts [5]. The study employed a flexible approach to interpret the data, combining inductive and deductive approaches, aligning with its exploratory nature to remain open to new insights while contextualizing findings within existing literature [16].

Four coders (XL, DV, MA, and ZL) conducted the analysis following Braun and Clarke’s [18] framework to systematically identify patterns, categories, and themes. Initially, all researchers reviewed the transcripts in depth. Each transcript was then coded by at least two researchers. A codebook was developed based on initial coding, and all transcripts were recoded using the finalized codebook. Themes were identified and refined through iterative discussions among all authors. Data analysis was facilitated by ATLAS.ti 23.

### Ethical Considerations

The study was approved by the Research Ethics Committee Faculty of Health, Medicine and Life Sciences of Maastricht University (FHML-REC/2023/124). Informed consent was obtained from all participants before their inclusion in the research. The research was conducted in accordance with the Declaration of Helsinki and its later amendments.

### Reflexivity

We critically reflected on how our identities influenced this research. XL focuses on assessment, SRL, and teacher education. DV specializes in instructional design and innovation. MA specializes in instructional design and e-learning. ZL focuses on simulation and technology in education. DD specializes in curriculum design. DV, MA, and DD teach in the MHPE program, whereas XL and ZL are not directly involved. This combination of internal and external perspectives may foster critical reflection, balance program-specific insights, and mitigate potential biases.

## Results

Analysis produced four key themes: (1) Providing feedback at the task level is easier than giving suggestions for further development of competencies; (2) Tailoring narrative feedback to personalized learning paths is challenging; (3) The primary role of coaching is to foster students’ reflection and autonomy, while adapting to students’ needs when necessary; (4) Coaching requires a balance between stimulating autonomy and fulfilling graduation requirements.

### Providing feedback at the task level is easier than giving suggestions for further development of competencies

Feedback providers consistently expressed a strong intention to support student learning, believing their feedback could help students become better learners. They felt confident in giving task-specific feedback but described facing a dilemma in delivering what one termed a “double message” (P5, FG2): making feedback contextual and meaningful while also aligning it with the broader competency framework. As one participant explained, “I struggle with balancing task-related and process-related feedback…the competencies and content are not separate. I want to give task-specific feedback on content while also offering process-oriented feedback that students can apply across tasks, but this is difficult” (P3, FG1).

They further noted that translating performance into the structure of the competency framework posed a recurring challenge. Feedback providers described the effort required to separate narrative feedback into distinct “boxes,” particularly when competencies overlapped or lacked clear definitions. One noted, “It becomes more of a sort of puzzle. I’m more thinking about: ‘Which box does this fit?’ rather than ‘What is the key message I want students to take away?’” (P16, FG5). Another reflected on a team discussion about this issue: “Some competencies proved to be overlapping. I hope we have fewer competencies and more clarity. Then it would be easier to follow up and offer feedback” (P12, FG4).

Feedback providers also struggled with moving from task-level feedback to competency-level judgments. While they found it relatively straightforward to comment on individual assignments, giving feedback on overall competency achievement or competency development was more complex when they were reviewing a single learning task. As one shared, “I find it difficult to distinguish: is this a moderate level or high level [of competencies]? And should I adapt my feedback to the type of assignment? That’s something we struggled with a lot” (P12, FG4).

### Tailoring narrative feedback to personalized learning paths is challenging

Feedback providers observed that elective tasks enabled students to pursue individualized learning paths, yet they found it difficult to tailor feedback without knowing more about those individual learning paths. They noted that they typically saw “only the learning task” from students, which limited their judgment on “what type of feedback would help most” (P16, FG5). They reported difficulty with suggesting next steps, as they often gave feedback on isolated tasks without insight into students’ prior performance or future goals. “We’re assumed to adjust feedback to competency levels, but it’s hard. I don’t have any clue about previous feedback or current levels,” one explained (P12, FG4). Another added, “I cannot always provide guidance…I simply do not know what students will do next” (P2, FG1).

The largely online nature of the program was an additional complication: “I simply do not know the students, so it’s really difficult to tailor your feedback” (P3, FG1). Feedback providers were unsure whether feedback was understood or useful. “I’m not listening to how students process this feedback…it’s really hard to reflect on how helpful it is” (P14, FG4). Another noted, “I really miss the feedback dialogue…I would prefer to have a conversation with the student about the work they handed in” (P2, FG1).

### The primary role of coaching is to foster students’ reflection and autonomy, while adapting to students’ needs when necessary

With regard to their coaching role, participants emphasized that they made students lead the coaching meetings, emphasizing their belief in students’ independence and capacity to take ownership. Coaches saw their role primarily as facilitators of reflection, gradually reducing support to encourage student autonomy. One participant noted, “Students are very high-achieving, motivated, and have a clear sense of what they want. Coaching is easy, and I don’t have to do much; it’s more gentle guidance along the way” (P8, FG3). Another described coaching as “facilitating reflection using non-directive principles…being a longitudinal assistant and even a friend, with the learner using that resource” (P1, FG1).

Some participants, particularly those more heavily involved in the program, reviewed narrative feedback ahead of coach meetings to help students interpret it. One shared, “I quickly go through feedback before meetings and ask myself what stands out. Then we compare notes during the session” (P4, FG2). Others preferred to let students lead, only stepping in when students asked for clarification: “If a student asks, ‘What do you think about this feedback?’ I ask them back first. If they say, ‘I don’t understand,’ then I help think it through, but not from the start” (P2, FG1).

Participants reported that they tried to adapt their coaching support to students’ individual needs, but also noted that some students hesitated to ask questions or stopped seeking support altogether. They recognized students’ struggles but found it challenging to know when and how to intervene. As one participant reflected, “Some students disappear for a long time, then suddenly need much more attention at the end. They realize, ‘This is my final chance,’ and then I have a lot of work” (P9, FG3).

### Coaching requires a balance between stimulating autonomy and fulfilling graduation requirements

When serving as coaches, participants recognized that many students began the program with clear objectives and aimed to develop in specific professional roles, though this was not true for all: “Some students had a clear vision when choosing electives, but others were less certain” (P5, FG2). While supporting students’ own goals, coaches also encouraged them to broaden their focus and explore beyond their initial interests to foster overall competency growth. “By doing some tasks, students found some interesting and others less so. I tried to challenge them to try different tasks” (P4, FG2). Another participant said, “If students follow their interests, they probably will follow the skills that they already have and perhaps fine-tune those skills. At the same time, we also want them to close gaps in other competencies” (P6, FG2).

When supporting students in selecting elective learning tasks, participants emphasized that they encouraged students’ planning and reflection, not only focusing on academic achievement but also taking a more holistic view of students’ development beyond the MHPE program: “We were learning to decide whether to guide students where we want or let them think where they should go. It’s more than academic support…I was like a life coach” (P5, FG2).

However, participants observed that students often prioritized addressing competency gaps to meet graduation requirements over pursuing personal interests. Coaches found students’ anxiety about competency scores and assessment outcomes frequently constrained elective choices toward the end of the program. As one participant noted, “We end up picking electives not truly guided by feedback, because students worry whether their competency scores are sufficient for the assessment committee. This undermines coaching conversations” (P8, FG3). Another explained, “Students say: ‘My interest is here, but my dashboard doesn’t show enough competency, so should I choose the one I am better at?’” (P9, FG3).

## Discussion

This study shows that faculty members believe that PA enhances learning, but they experience challenges and tensions in stimulating students’ SRL in practice. It is not easy to tailor feedback and coaching to meet students’ individual needs and to align coaching for SRL with graduation requirements.

This study highlights a significant challenge: while task-level feedback is relatively straightforward to deliver, providing feedback at the competency level is more complex. Consistent with Schut et al. [19], this study highlights that feedback serves dual purposes: improving task performance and supporting decision-making at the competency level. This study found that for feedback providers, complexity arises from the difficulty of translating feedback on learning tasks into insights on longitudinal competency development. These observations resonate with Moonen-van Loon et al. [13], who identified similar challenges in competency-based assessment contexts.

Feedback providers experience challenges in adapting their feedback to students’ individual learning needs. In line with Roze des Ordons et al. [20], faculty members often struggle to provide individualized feedback and feedforward. While existing literature underscores the importance of individualized feedback in cultivating self-regulatory skills [4], few studies have explored the practical challenges of delivering individualized feedback effectively. As Englander and Carraccio [21] argue, these challenges may stem from the lack of a longitudinal and continuous learning environment, which undermines feedback effectiveness. This study further identifies key factors that may disrupt the continuum: (1) learners’ autonomy in selecting individualized learning pathways, (2) feedback providers’ limited awareness of students’ background and learning progress, and (3) the absence of synchronous feedback dialogue in a largely online setting.

When serving as coaches, participants viewed their primary role as fostering students’ reflection and autonomy, though they adapted their support to students’ needs when necessary. Thus, the longitudinal interaction between students and coaches seems more suited for supporting students’ SRL competency. Coaches promoted students’ SRL and agency by posing thought-provoking questions that encourage reflection and foster ownership of learning. This finding aligns with Heeneman et al. [9], who argue that autonomy within PA enhances students’ agency and, consequently, their self-regulated learning [22]. However, the study also reveals that coaches adapt their approaches to address specific challenges when students struggle to fully self-regulate or resolve issues independently. Coaches varied in both their coaching styles and the extent to which they adjusted to students’ needs.

Coaches also reported that they sometimes struggled to align their support for SRL with graduation requirements. They recognized that students have to balance addressing competency gaps for graduation criteria with personal development from a lifelong learning perspective. This finding echoes previous research showing that graduation requirements can undermine the intended effectiveness of PA and coaching when performance metrics take precedence over SRL [23,24]. While high-stakes assessments are integral to PA, they often create tension between meeting external requirements and fostering authentic SRL. This tension may lead students to prioritize graduation requirements over SRL.

While this study provides insights into how faculty members perceive providing feedback and coaching to support students’ SRL in PA, the study has several limitations. First, the study exclusively focused on faculty members’ perspectives, which does not capture the actual support strategies they employ in practice. Discussions with students and direct observational studies could provide a more comprehensive understanding of these strategies. Second, the setting of this study was primarily online. Feedback providers gave narrative written feedback in e-portfolios without meeting students either online or in person, and coaches engaged with students both in person and online. Therefore, the findings may have limited transferability to other educational contexts.

This study suggests practical implications for the curriculum structure and the roles of feedback providers and coaches in the context of PA. Acknowledging that students should take the lead in directing their competency development highlights the need for the explicit inclusion of SRL in the curriculum as a competency to be further developed and assessed. This implies that the curriculum should provide appropriate learning opportunities and support. In settings where many faculty members are involved in providing feedback, coaches are better positioned to support the longitudinal development of SRL, help students aggregate feedback, and guide them in deciding on next steps in their learning path. Second, fostering dialogue between feedback providers and students is crucial to ensure that feedback is both appropriate and understood. One way to achieve this is by creating opportunities for synchronous oral feedback rather than relying solely on written feedback. Other possibilities include asking students to specify areas in which they seek feedback, encouraging them to reflect on previous feedback and how they have applied it in new learning tasks, and supporting them in contacting feedback providers to clarify feedback they did not understand. Third, while fostering autonomy is essential for stimulating SRL, coaches are suggested to develop a diverse repertoire of strategies to address the varying needs of students.

## Conclusion

The findings of this study indicate that feedback and coaching generally stimulate learning and SRL, but also highlight significant challenges for faculty, such as balancing task-specific feedback with broader competency development, managing the tension between granting autonomy and enabling learning and decision-making, and navigating the uncertainty around students’ personalized learning paths. Although PA enhances students’ learning by offering more autonomy and tailored support, faculty also need to address and overcome the challenges PA presents to effectively guide students in their learning journey.

## Appendix 1 Focus Group Interview Guide

### Introductory question: (5 min)

*Thank you so much for participating in this interview. This research is about how feedback and coaching stimulate students’ SRL in MHPE*.

1. Before we go into the interview, could you briefly introduce yourself?

**Transition question: (5–10 min)**

2. What was your experience as a feedback provider or a coach in the last academic year?

**Key questions (40–45 min)**

3. Providing feedback and coaching are assumed to enhance students’ learning. What is your perception? Why does it work or not? Can you give an example of how and why this might work or not work?
4. How and why does feedback and coaching enhance or hinder students’ planning? Can you give an example of how and why this might work or not work?
5. How and why does feedback and coaching enhance or hinder students’ monitoring? What is going well, and what requires improvement? Can you give an example of how and why this might work or not work?
6. How and why does feedback and coaching enhance or hinder students’ reflection? Can you give an example of how and why this might work or not work?

**Final question (5 min)**

7. Would you like to address aspects which are not been discussed yet?

*Thank you for your participation*.
